# Functional screening of willow alleles in Arabidopsis combined with QTL mapping in willow (*Salix*) identifies *SxMAX4* as a coppicing response gene

**DOI:** 10.1111/pbi.12154

**Published:** 2014-01-07

**Authors:** Jemma Salmon, Sally P Ward, Steven J Hanley, Ottoline Leyser, Angela Karp

**Affiliations:** 1 Rothamsted Research, HarpendenHertfordshire, UK; 2 Sainsbury Laboratory, University of CambridgeCambridge, UK

**Keywords:** *Arabidopsis*, allelic diversity, mutant rescue, *Salix*, QTL, strigolactone

## Abstract

Willows (*Salix* spp.) are important biomass crops due to their ability to grow rapidly with low fertilizer inputs and ease of cultivation in short-rotation coppice cycles. They are relatively undomesticated and highly diverse, but functional testing to identify useful allelic variation is time-consuming in trees and transformation is not yet possible in willow. Arabidopsis is heralded as a model plant from which knowledge can be transferred to advance the improvement of less tractable species. Here, knowledge and methodologies from Arabidopsis were successfully used to identify a gene influencing stem number in coppiced willows, a complex trait of key biological and industrial relevance. The strigolactone-related *More AXillary growth (MAX)* genes were considered candidates due to their role in shoot branching. We previously demonstrated that willow and Arabidopsis show similar response to strigolactone and that transformation rescue of Arabidopsis *max* mutants with willow genes could be used to detect allelic differences. Here, this approach was used to screen 45 *SxMAX1, SxMAX2*, *SxMAX3* and *SxMAX4* alleles cloned from 15 parents of 11 mapping populations varying in shoot-branching traits. Single-nucleotide polymorphism (SNP) frequencies were locus dependent, ranging from 29.2 to 74.3 polymorphic sites per kb. *SxMAX* alleles were 98%–99% conserved at the amino acid level, but different protein products varying in their ability to rescue Arabidopsis *max* mutants were identified. One poor rescuing allele, *SxMAX4D*, segregated in a willow mapping population where its presence was associated with increased shoot resprouting after coppicing and colocated with a QTL for this trait.

## Introduction

The best characterized and widely studied plant today is undoubtedly *Arabidopsis thaliana*, a small, short-lived, flowering annual which has been developed as a model organism for understanding the complex processes underlying plant growth and development. As research efforts intensify worldwide to meet the rising challenges of food and energy security, there is increasing interest in transferring knowledge from Arabidopsis to species of commercial relevance and particularly to those plants that are much less tractable to study. This is particularly true for many trees where studies on developmental processes can be very challenging, due to their large size, longevity and perennial growth cycle.

Willows (*Salix* spp.) are among the fast-growing tree species that are grown commercially as short-rotation coppice (SRC) to provide a renewable and sustainable source of biomass for bioenergy, due to their ability to produce high yields with low fertilizer inputs and their ease of propagation as vegetative cuttings (Karp and Shield, 2008; Karp *et al.*, 2011; Volk *et al.*, 2006). Willows were initially coppiced for basket-making, and breeding programmes aimed at genetic improvement for biomass are relatively recent. They are highly diverse, and the 350–500 species recognized (Argus, 1997) provide a rich germplasm resource for breeding. However, data on the genetic basis of many important developmental processes are limited due to the difficulty and effort required to assess phenotypic differences in the field, particularly in mature coppiced stands, and the fact that there is no robust method for transformation to validate gene function. Willows are also dioecious, highly heterozygous, and ploidies range from diploid to dodecaploid. A fully annotated genome sequence has not yet been published, but synteny between the willow genetic map and poplar genome has been demonstrated (Hanley and Karp, 2013; Karp *et al.*, 2011).

Many of the target traits for improvement in willow are complex and not well understood, even in poplar, which is closely related and has been developed as a model tree (Yang *et al.*, 2009). One such trait of fundamental importance is coppicing response, that is, the vigorous regrowth that occurs following the removal of the stems at winter harvest. Coppicing response is central to the suitability of all trees grown for biomass production in SRC cycles, as it enables sufficient biomass yield to be obtained within shorter time frames than conventional forestry (Keoleian and Volk, 2005). The number of resprouted branches also affects tree and canopy architecture and stem composition. Coppicing has also been shown to reinvigorate the plants, accelerating canopy development and shoot growth towards a theoretical maximum because of the increased number of shoots that resprout initially per coppiced stool and the changes in leaf size, specific leaf area and increased net photosynthesis associated with juvenile growth (Kauppi *et al.*, 1988; Sennerby-Forsse, 1995; Sennerby-Forsse and Zsuffa, 1995).

Despite the key importance of coppicing response, the genetic regulation of this process with respect to the control of bud and shoot behaviour has been the subject of very limited study in trees, although genetic differences in coppicing response have been reported (Dick *et al.*, 1998; Li *et al.*, 2012; Sennerby-Forsse and Zsuffa, 1995). Coppicing response involves the activation of pre-existing axillary buds (in the stool) that have been previously kept dormant by apical dominance, and, in contrast to trees, the mechanisms underlying these processes are very well characterized in Arabidopsis (Leyser, 2005, 2009). Of particular interest in this context are the Arabidopsis *More AXillary growth (MAX)* genes (*MAX1-MAX4*), which are known to regulate axillary bud outgrowth and branching through the production and signal transduction of strigolactones (SL) (Domagalska and Leyser, 2011).

*MAX* genes are conserved in both monocots and dicots. Independent identification of mutations includes *max* in *A. thaliana*, *ramosus* (*rms*) in pea (*Pisum sativum*), *decreased apical dominance* (*dad*) in petunia (*Petunia hybrida*) and *dwarf* (*d*) or *high-tillering dwarf* (*htd*) in rice (*Oryza sativa*) (reviewed by Domagalska and Leyser, 2011). Orthologues of *MAX* genes have also now been studied in kiwifruit (*Actinidia chinensis*) (Ledger *et al.*, 2010) and chrysanthemum (*Dendranthema grandiflorum*) (Dong *et al.*, 2013; Liang *et al.*, 2010).

Currently, six members of the SL pathway have been identified. MAX3/RMS5/HTD1/D17/SICCD7 encodes CAROTENOID CLEAVAGE DIOXYGENASE 7 (CCD7) (Booker *et al.*, 2005; Johnson *et al.*, 2006; Vogel *et al.*, 2010; Zou *et al.*, 2006) and MAX4/RMS1/DAD1/D10 encodes CCD8 (Arite *et al.*, 2007; Snowden *et al.*, 2005; Sorefan *et al.*, 2003). Both are involved in the production of SLs (Alder *et al.*, 2008; Auldridge *et al.*, 2006; Booker *et al.*, 2004; Schwartz *et al.*, 2004), and in Arabidopsis function up stream of MAX1, a cytochrome P450, CYP711A1 (Booker *et al.*, 2005; Scaffidi *et al.*, 2013). D27 (Lin *et al.*, 2009) and D14/D88/HTD2 (Arite *et al.*, 2009; Gao *et al.*, 2009; Liu *et al.*, 2009) have been identified in rice and recently analysed in Arabidopsis (Waters *et al.*, 2012a, 2012b). While the iron-containing protein D27 is involved in SL biosynthesis (Alder *et al.*, 2008), the α/β-fold hydroxylase D14/D88/HTD2 binds and cleaves strigolactone and may be involved in its perception and/or processing (Hamiaux *et al.*, 2012; Kagiyama *et al.*, 2013). MAX2/RMS4/D3 is an F-box protein involved in SL signal transduction (reviewed by Domagalska and Leyser, 2011).

We previously demonstrated that strigolactone affects willow bud activity in a very similar manner to Arabidopsis (Ward *et al.*, 2013). Furthermore, transformation rescue of Arabidopsis *max* mutants can be used as a platform to identify functional variation between willow *MAX* alleles (Ward *et al.*, 2013). In this present study, this approach was exploited to identify allelic variation underlying coppicing response. A large Arabidopsis mutant rescue screen was performed to test a series of 45 *Salix MAX* orthologous (*SxMAX1-SxMAX4*) alleles for putative functional variation. With a view to enabling downstream genetic analysis if required, the alleles included were cloned from parents of large willow mapping populations generated from crosses made based on phenotypic differences in shoot number and branching architecture. Here, we report the results of the mutant rescue screen, and the downstream analysis of alleles highlighted as potentially interesting. Segregation of one such allele, *SxMAX4D,* was subsequently found to be associated with the number of shoots that resprouted after coppicing in willow. These results demonstrate the power of using Arabidopsis to test large numbers of allelic variants for the identification of useful variation for further study in more targeted resource-intensive, field-based studies in trees.

## Results

### Isolation of willow *SxMAX* alleles

In total, 47 *SxMAX* alleles encoding unique protein products were cloned from the 15 parents of 11 willow mapping populations (Table1). For all four *SxMAX* loci, the majority of genotypes screened yielded two DNA sequences per diploid genome, with the exception of *SxMAX1* in parental lines S3, RES0453, RES0506 and RES0628; *SxMAX2* in S3, RES0453 and RES0506; *SxMAX3* in RES0655; and *SxMAX4* in S3, RES0655 and RES0663, for all of which only one allele was amplified. From partial sequencing data, SNPs were discovered in *SxMAX3* from RES0615, RES0506 and RES0901, but only one of the two alleles was successfully cloned and analysed in this study. No *SxMAX3* alleles from RES0099, RES0432, RES0453 RES1059, or *SxMAX4* alleles from RES0432 were cloned here due to project time constraints. Where only one allele was identified, it is likely that the *Salix* clone from which the genomic DNA was extracted is homozygous at that locus. This is particularly likely for S3, for which no other heterozygous markers have been identified in this genome region in previous studies in the K8 mapping population. In other cases, for example, RES0506 where only one allele was obtained for *SxMAX1* and *SxMAX2*, multiple amplification attempts, and the use of *Salix* primers designed to regions conserved among all other amplified *Salix* species, did not result in the discovery of a second allele. However, due to the potential divergence between the allele sequences within RES0506 (considered to be *S. caprea* L. *x S. cinerea* L. *x S. viminalis* L.), the possibility that a second allele failed to amplify under the range of conditions used cannot be completely ruled out.

**Table 1:** DNA source information and inferred protein alleles

DNA source	Parent	Clone name	MAX1	MAX2	MAX3	MAX4
S3	*S. viminalis* L. x (*S. viminalis* L. x *S. schwerinii*)	S3	B, B	A, A	A, A	B, B
R13	*S. viminalis* L. x (*S. viminalis* L. x *S. schwerinii*)	R13	B, C	A, B	A, A	B, B
RES0099	*S. triandra* L.	‘Semperflorens’	G, F	O, P	*	O, Q
RES0432	*S. daphnoides* Vill.	Fastigiate	B, B	G, H	*	*
RES0453	*S. aurita* L.	–	D, D	I, I	*	E, F
RES0506	*S. caprea* L. x *S. cinerea* L. x *S. viminalis* L.	–	B, B	A, A	J,*	M, N
RES0615	*S. schwerinii* Wolf	K3 Hilliers (WB 50 0 354)	B, C	J, K	I,*	R, S
RES0627	*S. viminalis* L. x *S. schwerinii* Wolf	910006 ‘Bjorn’	B, C	A, M	A, H	B, U
RES0628	*S. viminalis* L. x *S. schwerinii* Wolf	910007 ‘Tora’	C, C	A, N	A, F	B, T
RES0655	*S. viminalis* L.	Stone Osier	B, B	A, D	D, D	B, B
RES0663	*S. viminalis* L.	Pulchra Ruberrima	B, B	A, A	A, A	B, B
RES0674	*S. viminalis* L.	English Rod	B, B	A, D	A, A	B, D
RES0789	*S. purpurea* L. x *S. viminalis* L.	Ulbrichtweide	B, E	A, E	E, G	G, H
RES0901	*S. x alberti* (*S. integra x S. suchowensis*)	42/17	H, H	E, E	K,*	J, K
RES1059	*S. x fresiana* (*S. viminalis* x *S. repens*)	–	B, B	B, L	*	B, L

* Indicates not known.

### Sequence diversity within willow *MAX* genes

**Figure 1:**
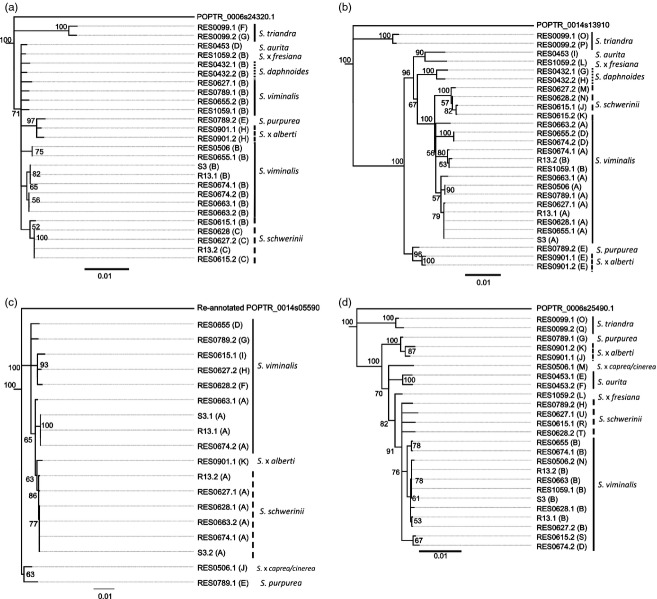
Consensus Trees of *Salix MAX* genes CDS with respective *Populus trichocarpa* orthologues. Rooted Bootstrap Consensus Trees (50% majority rule consensus) of *Salix MAX* genes CDS with *P. trichocarpa* orthologues used as the out-groups and bootstrap support node labels, generated from MUSCLE alignments (constructed using MUSCLE software available in Geneious Pro 5.5.6 (Drummond *et al.*, 2011) set to default settings, only *SxMAX3* needed minor manual adjustment so that clearly misplaced positions were more appropriately aligned) using Geneious Tree Builder software set to Jukes-Cantor; Neighbour-Joining; Boostrap Resampling with 100 replicates, also available in Geneious Pro 5.5.6 (Drummond *et al.*, 2011). (a) *MAX1*, (b) *MAX2*, (c) *MAX3*, (d) *MAX4*.

Analysis of the predicted coding sequences (CDS) for each locus revealed SNP frequencies of 33.3, 74.3, 29.2 and 53.8 SNPs per kb for *SxMAX1*, *SxMAX2*, *SxMAX3* and *SxMAX4*, respectively, and indicated that, on average, approximately one-third of the SNPs detected would result in nonsynonymous amino acid substitutions (Table2). *SxMAX2* alleles contained proportionately 30% or more SNPs than the other three loci (Table2). Analysis of CDS alignments (data not shown) and phylogenetic trees (Figure1) revealed that polymorphisms within all four loci were not evenly distributed between species but were concentrated within particular alleles. For example, alleles from *S. triandra* contributed a greater proportion of allelic difference to the loci as a whole, as revealed by their distance relative to other alleles in *SxMAX1*, *SxMAX2* and *SxMAX4* CDS trees (Figure1a, b and d). The separation of this species from the others indicated by these results is in agreement with previous *Salix* molecular diversity data reported by Trybush *et al*. (2008) as was the consistency of the allele clustering in accordance with current *Salix* taxonomic thinking. The latter was also true for alleles of hybrid genotypes, which were assigned to separate clusters based on their parentage. For example, alleles from RES0789 (a *S. viminalis x S. purpurea* hybrid) were consistently assigned to different groups as expected (Figure1a, b and d).

**Table 2:** Inferred CDS and protein information

Gene	*MAX1*	*MAX2*	*MAX3*	*MAX4*
Number of DNA sources	15	15	11*	14†
CDS length (bp)	1593	2085	1842, 1845, 1848, 1851	1674
Total number of SNPs per alignment‡	53	155	54	90
SNP frequency (SNPs per kb)	33.3	74.3	29.2§	53.8
Number of 3 bp INDELs	0	0	3	0
Number of nonsynonymous SNPs‡(% of total SNPs)	16 (30.2)	52 (33.6)	20¶ (37.0)	31 (34.4)
Alleles at inferred CDS level	16	22	11	21
Number of inferred protein sequences	7	14	9	17
Protein length (amino acids)	530	694	613, 614, 615, 616	557
Predicted% Identical Sites	97.0	92.5	96.3	94.4
Predicted Protein% Pairwise Identity	99.5	98.6	99.1	98.9

* Including RES0615, RES0506, RES0901 from which only one of two alleles are known and excluding RES0099, RES0432, RES0453 and RES1059.

† RES0432 not included.

‡ Excluding INDELs.

§ 1846.5 bp average length was used to calculate SNP frequency for MAX3.

¶ Excluding 3 bp indels.

**Figure 2:**
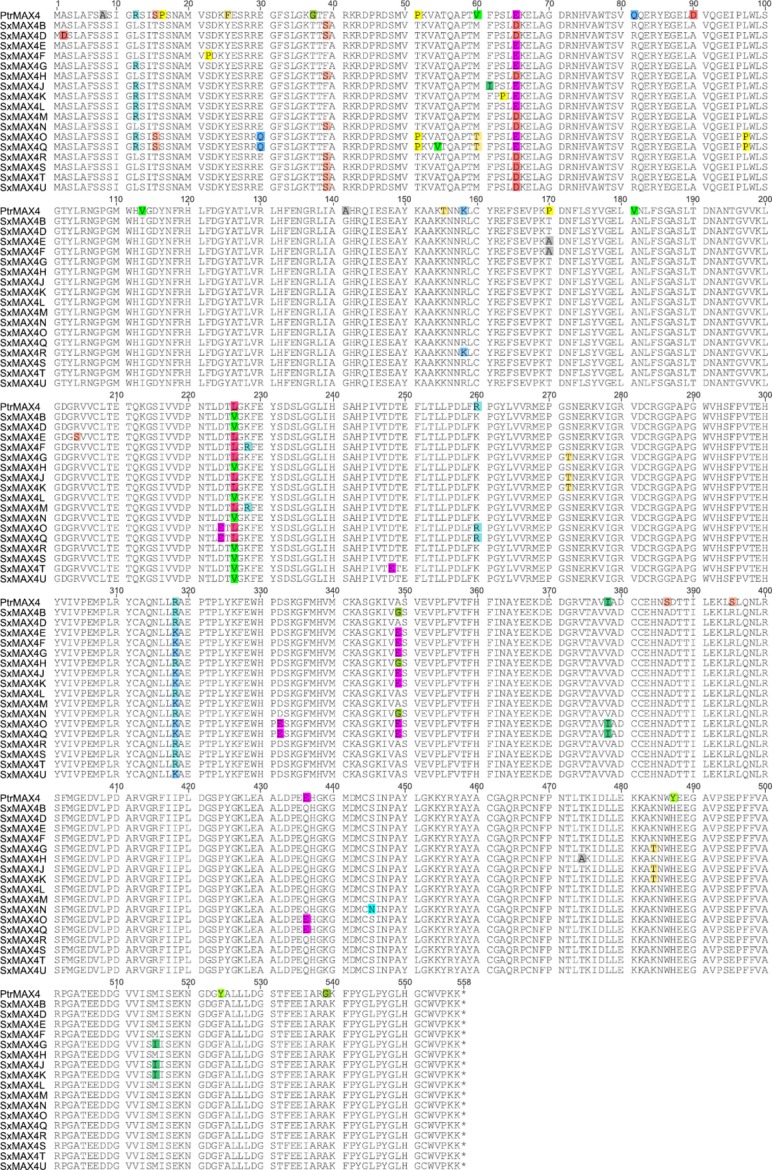
*Salix* and *Populus trichocarpa* orthologous protein alignments for MAX4. Inferred CDS were translated using Geneious Pro 5.5.6. (Drummond *et al.*, 2011). The inferred protein sequences of the *SxMAX* alleles were then aligned with the orthologous *P. trichocarpa* protein sequence using the MUSCLE algorithm within Geneious Pro 5.5.6 with default settings. Nonconsensus residues were highlighted according to Geneious Pro 5.5.6 default settings.

The inferred protein sequences of the *Salix* alleles aligned with the orthologous *Populus trichocarpa* sequences for *SxMax4* are shown in Figure2, while sequences for the *SxMAX1-3* loci are provided in Figure S1. The willow MAX alleles were 98%–99% identical, but a number of amino acid changes were identified (Figure2 Table2, Figure S1). Conserved positions in synapomorphic regions found in *MAX2*, *MAX3* and *MAX4* by Challis *et al*. (2013) were also conserved in all the *Salix* alleles. There were also no differences within the *Salix* alleles in any residues that correlated with previously published mutations, nor the proposed substrate specificity residues of *MAX3* and *MAX4* (Delaux *et al.*, 2012). Nonconservative changes were identified at three amino acid sites (Figure2) of which two were unique and therefore of potential interest. SxMAX4D (*S. viminalis*) has an alanine (neutral–nonpolar) to aspartic acid (acidic) substitution at position two. This alanine is highly conserved in other genera for which MAX4 protein sequence is available (Challis *et al.*, 2013). SxMAX4E (*S. aurita*) has an arginine (basic) to serine (neutral–polar) substitution at position 204 (position 214 of Supplementary data file 1 in Challis *et al.*, 2013). This arginine is present in all other genera studied. SxMAX4E carried several additional changes (Figure2) and has been reported previously (Ward *et al.*, 2013).

### Functional testing of *Salix MAX* alleles

**Figure 3:**
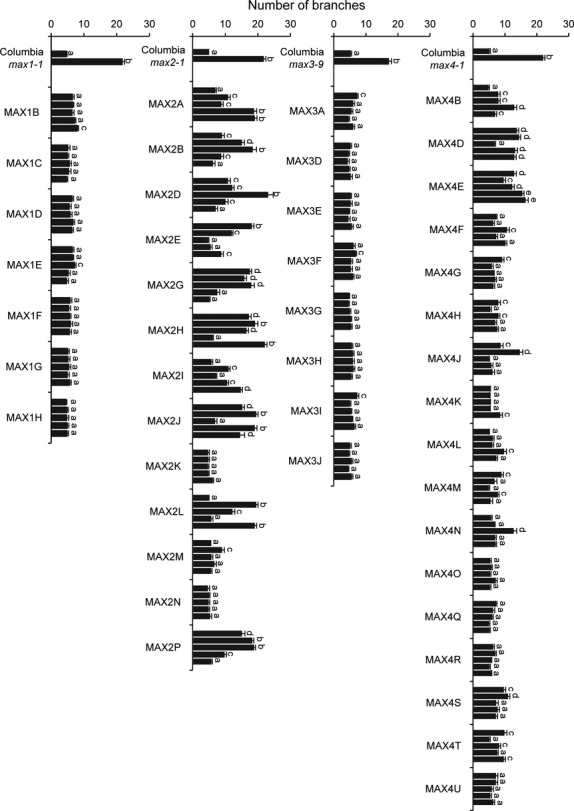
Testing allelic variation in *SxMAX* alleles through transformation rescue of their cognate Arabidopsis *max* mutant. For each *MAX* gene, branch numbers were determined for ten independent transgenic lines for each of the *Salix* alleles isolated. Branching assays were as described by Greb *et al*. (2003). For each allele, data are shown for five lines representative of the full data range, with wild-type *Columbia* and the corresponding *max* mutant as controls. Data represent the mean number of branches ± standard error (*n* = 15–20). The different letters denote significantly different means (*P* < 0.05) as determined using Tukey's test. Branching data from lines *MAX4B*, *E* and *G* have previously been published in Ward *et al*. (2013).

Forty-five willow alleles were transformed into their cognate Arabidopsis *max* mutant, and branch numbers ascertained in the resultant transgenic lines. Ten independent lines per construct were taken to homozygosity and assayed. The data from five lines are shown, representing the full range of phenotypes observed (Figure3). Multiple independent transgenic lines were assayed for each construct to account for the fact that different transgenic lines carrying the same construct can show quantitative differences, due to transcriptional or post-transcriptional effects (Schubert *et al.*, 2004). Branching data from lines containing *MAX4B*, *E* and *G* have previously been published in Ward *et al*. (2013) but are included here again for comparison.

All of the *SxMAX1* and *SxMAX3* constructs were able fully to rescue *max1* and *max3* shoot branching to a wild-type level, and little variation was observed between the independent transgenic lines (Figure3). In contrast, a great deal of variability in rescue ability was observed between independent transgenic lines transformed with *SxMAX2* constructs (Figure3). For each construct, however, at least one line was able to restore branching of *max2-1* to wild-type levels (significance tested by Tukey test), demonstrating that they all had the ability to complement the *max2* phenotype. Of the seventeen *SxMAX4* constructs transformed into *max4-1*, fifteen showed wild-type branching among the majority of the independent transgenic lines (Figure3). All five of the *SxMAX4E* lines and four of the five *SxMAX4D* lines presented in Figure3 (which had unique amino acid differences at positions 204 and 2, respectively) only partially reduced *max4-1* branching.

**Figure 4:**
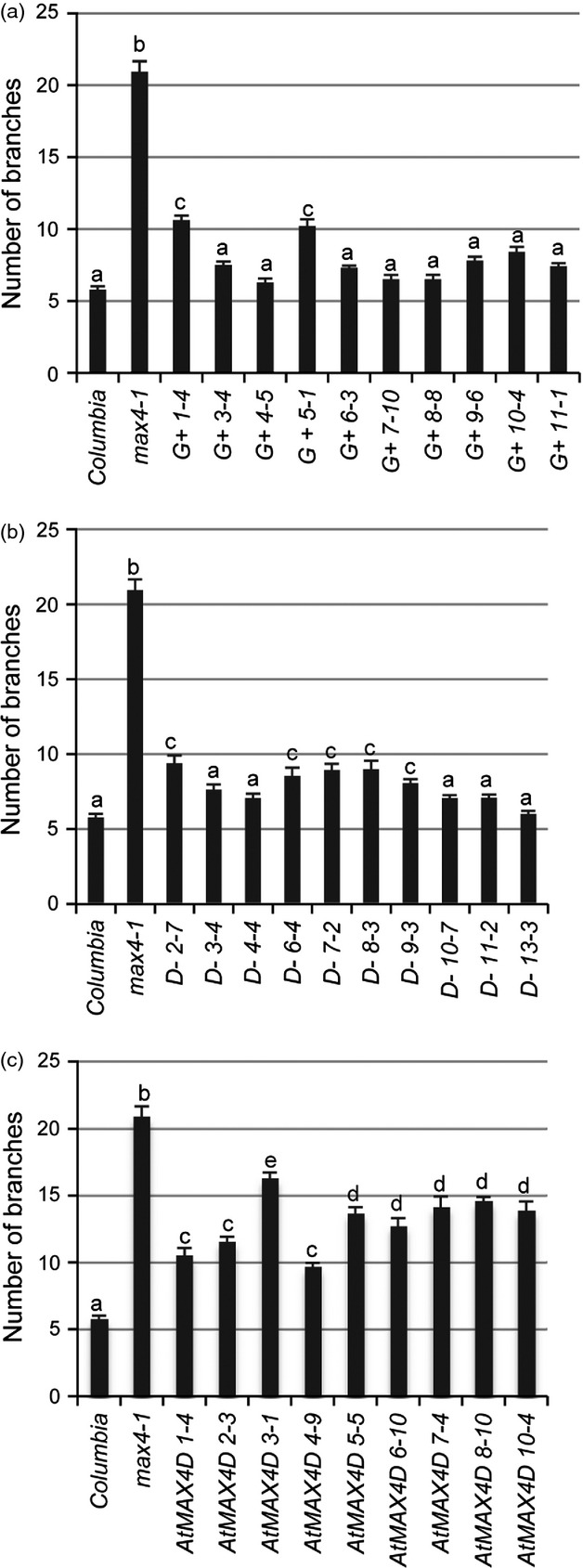
Testing the influence of amino acid position two in *SxMAX4* through rescue of *max4-1* branching. Mean branching levels in 10 independent lines of *max4-1* transformed with (a) SxMAX4G+, in which the alanine at position two of a fully rescuing SxMAX4G was mutated to aspartic acid; (b) SxMAX4D-, in which the aspartic acid at position two of SxMAX4D, which is only able partially to reduce *max4-1* branching, was mutated to alanine; (c) AtMAX4D, in which the alanine at position two of the fully rescuing *Arabidopsis* MAX4 (Sorefan *et al.*, 2003) was mutated to aspartic acid. Branching was assayed as described by Greb *et al*. (2003). Branch numbers were determined in 9–10 independent transgenic lines for each construct, with wild-type *Columbia* and *max4-1* as controls. Data represent the mean number of branches ± standard error (*n* = 15–20). The different letters denote significantly different means (*P* < 0.05) as determined using Tukey's test.

To test whether the substitution of the conserved alanine at position two to aspartic acid in SxMAX4D was responsible for the partial rescue of *max4-1* branching by the *SxMAX4D* allele, a number of constructs were made in which: (i) the alanine at position two of SxMAX4G (an allele able to rescue fully *max4-1*) was mutated to an aspartic acid (SxMAX4G+); (ii) the aspartic acid at position two in SxMAX4D (an allele unable to rescue fully *max4-1*) was mutated to alanine (SxMAX4D-); and (iii) the alanine at position two in Arabidopsis *MAX4* (which can fully rescue Arabidopsis mutant *max4-1* (Sorefan *et al.*, 2003) was mutated to aspartic acid (AtMAX4D). All three constructs were transformed into *max4-1,* and ten homozygous independent lines tested for their ability to rescue the highly branched *max4-1* phenotype. Lines expressing *SxMAX4D-* (aspartic acid changed to alanine) were able fully to rescue *max4-1* branching in an increased number of lines (compare Figure3 with Figure4c). In the case of *AtMAX4D* (alanine changed to aspartic acid), none of the ten independent transgenic lines studied were able fully to rescue *max4-1* (Figure4c). These data suggest that the alanine at position 2 is important for MAX4 function. However, SxMAX4G+ (alanine changed to aspartic acid) was unchanged in its ability to restore wild-type branching as compared to SxMAX4G (compare Figure 3 with Figure 4a), indicating that while important, the alanine is not essential in some allelic contexts.

### Functional relevance in willow

**Figure 5:**
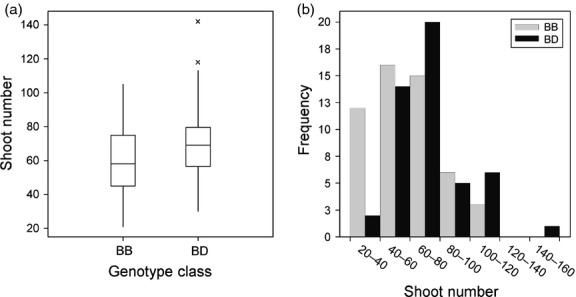
Testing functional relevance in a willow mapping population. (a) Boxplot showing significant difference (*t *=* −*2.76, *P* = 0.007; Student's *t*-test) between *SxMAX4* BB and BD classes for the number of resprouting shoots postcoppice (mean BB 59.10, mean BD 71.31; SED = 4.419; d.f. = 98) for 100 randomly selected individuals of the mpA willow mapping population. (b) Frequency distributions for shoot number postcoppice for BB and BD classes.

**Figure 6:**
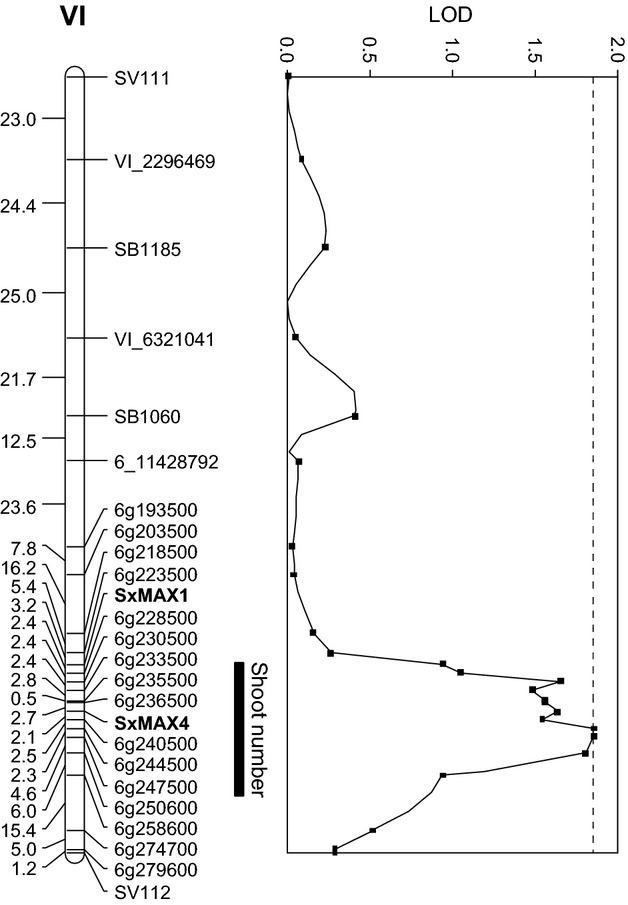
A significant QTL for shoot number production after coppicing colocates with segregation of the *SxMAX4D* allele. The parental linkage map spanning chromosome VI for *S. viminalis* genotype RES0674 is shown. Markers are SNPs or microsatellites (prefixed SB- or SV-). Markers names beginning with *6 g* represent orthologous genes in the *Populus* genome sequence (www.phytozome.net). The QTL locates with segregating *SxMAX4B* and *SxMAXD* alleles, which also showed differential mutant rescue capability in Arabidopsis *max4* mutant lines. The QTL length reflects a 1-LOD decrease from the peak position and excludes *SxMAX1*. The chromosomal significance threshold is indicated by the dashed line.

One of the available willow mapping populations, mpA, has a suitable genotypic constitution (expected 1 : 1 pseudo testcross segregation) to test whether any coppicing phenotypes corresponded with *SxMAX4D* segregation. From this population, 100 genotypes were randomly selected and assessed for resprouting from the coppiced stool. After excluding the possibility of spatial trends in the field data, a significant difference in the number of resprouted stems was detected in this population according to the presence/absence of the *SxMAX4D* allele (*P* < 0.01; Student's *t*-test) (Figure 5). Subsequent QTL analysis by interval mapping detected a significant QTL on paternal (RES0674) linkage group VI that colocated with segregating alleles *SxMAX4B* and *SxMAX4D* (Figure 6). The QTL explained 8.2% of the phenotypic variance. At the population level, plants heterozygous for *SxMAX4B* and *SxMAX4D* alleles produced a greater number of stems than those homozygous for *SxMAX4B*.

## Discussion

### Allelic diversity in the mapping populations

Our data provide a detailed survey of allelic diversity at the *SxMAX1*, *SxMAX2*, *SxMAX3* and *SxMAX4* loci in 15 parents of *Salix* mapping populations used in the willow breeding programme at Rothamsted Research, UK. Currently, breeders of biomass willow in Europe tend to select lines containing *S. viminalis,* and the Rothamsted populations reflect this bias (Table1). There are few data available on allelic diversity in willow breeding stocks, but, within the set used here, our data indicate that SNPs were present at a frequency of between 29 and 74 per kb in the exons of the MAX genes. Although several species other than *S. viminalis* are present in this set, either as pure species or in the pedigrees of the bred lines, the majority of the diversity found arose from the divergence of *S. triandra* RES0099 from the other species (as highlighted by a distinct node in the CDS phylogenetic trees in Figure 1). This is consistent with the findings of Trybush *et al*. (2008), who first noted the separation of *S. triandra* from a wide range of other *Salix* species. Our findings are within a range reported for *Salix* exons in a recent study in which between 8 and 96 SNPs/kb were detected among 12 genes involved in wood formation for a diverse range of *Salix* taxa (Perdereau *et al.*, 2013). In poplar, from 8 to 105 SNPs were detected per kb, depending on the species and genes investigated (see also Perdereau *et al.*, 2013). These data suggest that SNP frequencies are generally high for the *Salicaceae*, in comparison with those for ESTs of wheat (16.5) and maize (8.9), for example (Barker and Edwards, 2009). The genus *Salix* comprises a large range of diverse species, over 10-fold more than *Populus*. *MAX* genes are conserved among genera, but our findings suggest there is a high probability of uncovering further novel allelic diversity of use to breeders if a wider number of species is examined.

We found considerable variation between the genes examined in the extent of allelic diversity. The short branch lengths of the *MAX1* phylogenetic tree indicate that the *SxMAX1* alleles contain between them fewer base substitutions per site relative to the other three loci in these willow species. In contrast, even taking into consideration the longer CDS for *SxMAX2*, alleles at this locus contained between them at least 30% or more SNPs than the other three loci. However, in the *Salix* individuals studied here, the diversity occurred within relatively few alleles compared with the other loci, such that total numbers of alleles were not higher for *SxMAX2* than *SxMAX4*, for example, where polymorphisms were more evenly spread.

### Functional significance of allelic variation

In a proof-of-principle study, we have previously shown that it is possible to detect functional allelic variation in the coding sequence of willow *MAX* genes using transformation rescue of the cognate Arabidopsis mutant. The data presented in this study provide a much more extensive survey of the functional allelic variation in the *MAX* gene coding sequences of the Rothamsted mapping population parents. Of the 45 alleles tested, only two showed evidence of consistently poor rescue, implying that most of the allelic diversity in the alleles does not affect gene function to the degree detectable in this assay. The sensitivity of the assay is difficult to judge, but it is important to note that the degree of variability between the independent transgenic lines was highly dependent on the transgene, which will affect the ability to detect functional allelic diversity above the noise of variability in transgene activity. It is therefore possible that some functional diversity, for example among the MAX2 alleles, was missed. At the opposite end of the spectrum, it is possible that very low levels of activity from, for example MAX3, are sufficient for full rescue, which might also reduce the sensitivity of our assay. These factors make it likely that not all the functionally important variation in the alleles was detected in this study, and certainly, transforming with coding DNA from start to stop codons only, as carried out here, would be unlikely to identify polymorphisms affecting expression level. Nonetheless, it is interesting that the alleles showing evidence of reduced function were those with nonconservative substitutions in conserved amino acids, suggesting that this could be used to prioritise allele testing with relatively low risk of missing important variation.

### MAX4D

The results obtained here suggest that the presence of an aspartic acid residue at position two, or the absence of an alanine, can reduce the ability of MAX4 to function. The consensus residue at this position is alanine, and when it is replaced with aspartic acid, as in SxMAX4D, ability to rescue the Arabidopsis *max4-1* is reduced, while substituting the aspartic acid for an alanine, thereby restoring the consensus, improves the ability of the allele to rescue. Furthermore, a mutated Arabidopsis MAX4 protein in which the alanine to aspartic acid change was replicated could no longer fully rescue the Arabidopsis *max4-1* branching phenotype. Taken together, these data suggest that the alanine at position 2 is worthy of further study with respect to MAX4 function. However, changing the same alanine in SxMAX4G to aspartic acid had no effect on the ability of the resulting protein to rescue the *max4-1* branching phenotype. Examination of the protein sequences (Figure 2) shows additional changes are present in SxMAX4G compared with SxMAX4D, suggesting that the alanine is not required and/or the aspartic acid is not detrimental in all allelic contexts.

### MAX4D and the willow coppice response

Previous studies have shown that mutant lines of Arabidopsis can be used to test functional differences between orthologous genes from commercially important plants based on their ability to rescue cognate mutant lines (Ward *et al.*, 2013). This study progresses this a further step and associates the allelic differences with regulation of a complex trait. The MAX4D allele was found to be associated with increased resprouting after coppicing, and the MAX4 locus was found to map within a QTL for this trait. These data suggest that a component of variation for a highly complex trait has been resolved to the level of single candidate quantitative trait nucleotide (QTN) – a step rarely achieved in quantitative genetic studies in crop plants.

Prior to this study, a genetic basis to coppicing response had not been identified. The finding that the *MAX4* locus plays a role now enables manipulation of this trait, which has diverse relevance in terms of applications. Coppicing reinvigorates growth and an improved coppicing response would be beneficial for improving productivity of SRC systems (Kauppi *et al.*, 1988). Stems in coppiced stands also have higher bark proportions compared with single stem systems as the bark is roughly proportional to the area:volume ratio of the stem (Adler *et al.*, 2008). This has relevance for the use of wood chip in thermal conversion technologies where alkali metals (e.g. K and Na) in the bark can cause problems and where the nitrogen or sulphur containing compounds form NOx or SOx in the exhaust gases. Conversely, bark is a source of extractives comprising many diverse compounds (Kammerer *et al.*, 2005) and may come to have value in biorefining as source of valuable bioproducts and/or industrial chemicals. Manipulation of bark proportions by managing the numbers of shoots that resprout after coppicing provides a novel route to optimizing SRC feedstock for different industrial uses. Selections based on allelic diversity at *MAX4* will now be incorporated into the breeding programme at Rothamsted, UK.

Previous research on coppicing in willow has investigated the histology of bud activation (Sennerby-Forsse and Zsuffa, 1992, 1995) and focussed on applied aspects, mostly on finding the optimal balance between the positive effects of coppicing and the negative effect of leaving insufficient time between harvests for the build-up of remobilised reserves in the stool and roots (Verwijst, 1996a, 1996b). It is well known that shrubby willows (subgenus *Vetrix*) respond more vigorously to coppicing than tree willows (subgenus Salix), suggesting a genetic basis to this trait. However, now that the importance of *MAX4* has been identified, future studies can focus on understanding the role of strigolactones in coppicing response and on determining the mechanisms regulating final shoot numbers in coppiced stools.

Success in mapping a QTL for coppicing response in this study was dependent on the method of scoring the phenotype with respect to number of shoots. Previous studies have reported QTL for stem traits (Hanley and Karp, 2013; Tsarouhas *et al.*, 2002), but shoot number in willow can be difficult to resolve by QTL mapping because it is normally scored at the end of each year and/or at harvest (i.e. at the end of the 3-year coppice cycle). However, the shoot number of mature willow plants is not simply the product of the number of shoots that initially sprouted from the stool. Self-thinning of the sprouted shoots also occurs in which many buds sprout simultaneously, and the resultant stems are then progressively thinned to a lower number. This self-thinning process is thought to involve differential growth rates, dominance and suppression of stems and re-allocation of resources (Sennerby-Forsse *et al.*, 1984). The proportion of buds that sprout and the degree of self-thinning both vary among genotypes, but the nature of the relationship between them has yet to be resolved. In recognition of the complexity of this trait, the approach used here was to count the number of shoots that sprouted from the cut-stool as an indicator of coppicing response instead of shoot number at harvest. Studies aimed at understanding the relationship between self-thinning and the proportion of activated and sprouted shoots are now underway.

Willow has been the focus of the current study, but the results on coppicing response have relevance to other tree species, including poplar, and the approach adopted could be applied to investigate other candidate regulators of agronomically important complex traits. Genetic crosses are resource intensive to phenotype, particularly in large trees, and prior knowledge of functional diversity will enable efforts to be targeted to those crosses segregating for specific alleles of interest.

## Materials and methods

### Willow material

The *Salix* plants comprised a set of confirmed diploid genotypes from the NWC that were chosen on the basis of phenotypic differences in branch number and architecture as parents of mapping populations for a wide diversity of trait studies. The populations were planted between 2008 and 2009 and are maintained at Rothamsted Research (RRes). Details on the genetic background of these genotypes are provided in Table1 and in Hanley and Karp (2013). Genomic DNA was extracted using DNeasy Plant extraction kit (Qiagen, Crawley, UK).

### Isolation of the *Salix MAX* genes

Putative orthologues of the *Arabidopsis MAX1* (*At2 g26170*), *MAX2* (*At2 g42620*), *MAX3* (*At2 g44990*) and *MAX4* (*At4 g32810*) genes were identified in the *Populus trichocarpa* v2.2 genome sequence (http://www.phytozome.net) as described in Ward *et al*. (2013). *Salix* orthologues of the *P. trichocarpa MAX1 (POPTR_0006s24320)*, *MAX2 (POPTR_0014s13910)*, *MAX3 (POPTR_0014s05590)* and *MAX4 (POPTR_0006s25490)* genes were then amplified, initially from the diploid genotype R13 [*S. viminalis x* (*S. viminalis x S. schwerinii*); (Hanley *et al.*, 2006)], using primers designed to predicted *P. trichocarpa* coding sequence (CDS). Resulting PCR products were gel-purified (QIAquick Gel Extraction Kit, Qiagen), sequenced and mapped on a *P. trichocarpa*-anchored *Salix* map (K8; Hanley *et al.*, 2006) to confirm orthology (Ward *et al.*, 2013). *Salix* sequences were assembled using ContigExpress in Vector NTI 10.1.1 (Life Technologies Ltd, Paisley, UK).

*SxMAX* alleles were cloned from the set of diploid *Salix* genotypes that are parents of mapping populations maintained at RRes (Table1). Amplifications used genomic DNA as a template and *Salix*-derived primers (Table S1). For S3 and R13 (parents of the K8 mapping population), primers designed to the 5′ and 3′ CDS ends of each gene were used. To account for known polymorphisms at equivalent CDS priming sites in the other genotypes studied, primers were designed outside the CDS for *SxMAX1*, *SxMAX2* and *SxMAX4* (Table S1). Increased diversity in the *SxMAX3* upstream sequence prevented use of a common primer in this region. Instead, several genotype-specific 5′ CDS primers were used in conjunction with a common 3′ CDS primer (Table S1). AccuPrime Pfx SuperMix (Invitrogen) was used to generate PCR products that were gel-purified, cloned into the pENTR/D-TOPO vector (Invitrogen) and sequenced to obtain coverage of all exons. To ensure sequences analysed were true to the genomic DNA, either multiple individual clones were sequenced, or clones were compared with corresponding genomic sequence generated by direct sequencing of PCR products or both. BLASTN searches against the *P. trichocarpa* v2.2 genome sequence were used to ensure that all cloned sequences showed a best hit to the original *P. trichocarpa* orthologous gene target and not a paralogous sequence.

### Sequence analysis

*Populus trichocarpa* v2.2 gene models (www.phytozome.net) were used to infer *SxMAX* CDS sequences. *MAX2* has no introns in any species so far examined. Examination of the predicted splice sites for *MAX1, MAX3* and *MAX4 P. trichocarpa* indicated that, with the exception of one intron within *MAX3*, the terminal GT and AG residues of all predicted introns were fully conserved between *Salix* and *P. trichocarpa*. The discrepancy in *MAX3* (in which the A of the predicted *P. trichocarpa* 3′ AG splice site of intron six is consistently a G in *Salix*) suggested that the predicted *P. trichocarpa* gene model may be incorrect, with an extra intron predicted. Although a paucity of public *Salix* or *Populus* mRNA/EST sequences for this gene prevented a more definitive analysis, comparison with *MAX3* orthologous proteins from several other plant genera also suggested the extra *P. trichocarpa* intron is erroneous. Therefore, cDNA of this region of *POPTR_0014s05590* from *P. trichocarpa* (RES 309/01 ‘Manmillan’) was sequenced and did not contain the sixth intron, confirming the *P. trichocarpa* v2.2 prediction was erroneous. The CDS sequence based on a *P. trichocarpa MAX3* gene model, but omitting the predicted sixth intron annotation, was used throughout this study.

Nucleotide sequences were aligned using the MUSCLE algorithm within Pro 5.5.6 software (Drummond *et al.*, 2011; Kearse *et al.*, 2012) using default settings. Only *MAX3* required some minor manual adjustment. Alignments were then scored for synonymous and nonsynonymous SNPs. The MUSCLE alignment for each *MAX* locus was used to construct a rooted phylogenetic tree with the corresponding *P. trichocarpa* CDS as an out-group (Geneious Tree Builder default settings; cost matrix: 65% similarity (5.0/−4.0), Genetic Distance Model: Jukes-Cantor, Tree build Method: Neighbour-Joining; Bootstrap Resampling 100 replicates).

Inferred CDS were translated using Geneious Pro 5.5.6. (Drummond *et al.*, 2011). The inferred protein sequences of the *SxMAX* alleles were then aligned with the orthologous *P. trichocarpa* protein sequence, again using the MUSCLE algorithm within Geneious Pro 5.5.6 with default settings. Only SxMAX3 needed minor manual adjustment.

### Generation of transgenic Arabidopsis

The *SxMAX* alleles were transferred from pENTR/D-TOPO to the GATEWAY compatible binary destination vector pK7WG2 (Karimi *et al.*, 2002) using LR Clonase II Enzyme Mix (Invitrogen) and transformed into the corresponding Arabidopsis *max* mutant background via *Agrobacterium tumefaciens* strain GV3101 using the floral dip method (Clough and Bent, 1998).

All Arabidopsis *max* lines are in the Columbia-0 background. For transformation, *max1-1*, *max2-1, max3-9* and *max4-1* plants were grown in 8-cm square pots containing F2 compost treated with Intercept 70WG (both from Levington Horticulture, Ipswich, UK). The plants were grown in a growth room at 21 °C with 16 h light, 8 h dark and a light intensity of 100–120 μmol/m^2^s^2^.

Transformants were selected on agar solidified ATS medium containing 50 μg/mL kanamycin (Sigma, Poole, UK). For each construct, at least 10 independent single insertion lines were taken to homozygosity.

### Shoot branching assay

A decapitation assay was used to quantify branching (Greb *et al.*, 2003; Ward *et al.*, 2013) in which the total number of rosette branches produced following decapitation were scored. Plants grown in P40 multitrays (Desch Plantpak Ltd, Maldon, Essex, UK) in F2 compost as above. Five of the ten independent single insertion lines studied have been chosen to reflect results for each allele and are presented graphically.

### Mutagenesis

To produce the *SxMAX4D-* construct, the aspartic acid at position two in *SxMAX4D* was mutated to alanine by amplification from the pENTR/D-TOPO vector containing *SxMAX4D* using the forward primer CACCATGGCTTCCTTGGCATTTTCC, which replaced the A at CDS base five with a C, in conjunction with the common reverse primer located outside of the Stop codon GATAGCTAAATCACACAACCCC. The *SxMAX4G+* construct was generated using the same approach, but by amplification from the *SxMAX4G* construct using the forward primer CACCATGGATTCCTTGGCATTTTCCTC which replaced the C at CDS base five with an A and therefore an alanine at position two with an aspartic acid. The common reverse primer GATAGCTAAATCACACAACCCC was used. The mutated alleles were fully sequenced for verification as described in ‘Isolation of the *Salix MAX* genes.’

To change the alanine at position two to aspartic acid for the generation of an Arabidopsis based version of *SxMAX4D* (*AtMAX4D*), the CDS of *MAX4* was amplified from Columbia cDNA using the forward primer ATCCCGGGATGGATTCTTTGATCACAAC which replaced the fifth base of the CDS with an A instead of a C, and reverse primer GATCTAGATTAATCTTTGGGGATCCAGCA. Restriction sites are present in the primers to clone the *AtMAX4D* amplicon behind the 35S promoter in the pART7/pART27 vectors (Gleave, 1992).

### QTL analysis

Segregation of *SxMAX4D* was tested for phenotypic association with coppicing response in willow mapping population A (mpA), which was planted in a field experiment at Rothamsted Research, Harpenden, Hertfordshire, UK, in March 2008. The male parent of the mpA population (*S. viminalis* RES0674) is heterozygous for SxMAX4D and SxMAX4B alleles, while the female parent (K8-411) is homozygous SxMAX4B. Following establishment growth after planting in Spring 2008, plants were first coppiced in February 2009 and then again in February 2011 after 2 years’ uninterrupted growth. In Spring 2011, the total number of stems resprouting from stools was recorded for a random set of 100 mapping population individuals and 11 clonal replicate controls (genotype K8-411) regularly spaced throughout measured area. Mixed modelling was used to test for any spatial trends across the field site but none were found. Trait data were tested for association with SxMAX4 genotype using a Student's *t*-test. QTL analysis was then used to determine the chromosomal position of the underlying variation. A genetic map of chromosome VI was generated by screening a set of previously developed microsatellite and SNP markers against 384 mpA progeny, using genotyping methods previously described by Hanley *et al*. (2006). Interval mapping within MapQTL 4.0 software (Kyazma®, Plant Research International, Wageningen, the Netherlands) was used for initial QTL analysis. To maximize resolution once a rough QTL position was identified, additional SNP markers in the vicinity of *SxMAX4* were developed and mapped as before, and the QTL analysis repeated. A chromosomal QTL significance threshold was determined by permutation test (1000 permutations).

## Supporting Information

Figure S1 *Salix* and *Populus trichocarpa* orthologous MAX protein alignments for MAX1-3.

Table S1 *Salix MAX* cloning information.
