# Supplementary material for: Functional screening of willow alleles in Arabidopsis combined with QTL mapping in willow (Salix) identifies SxMAX4 as a coppicing response gene
Source: Plant Biotechnol J. 2014 Jan 7;12(4):480–91. doi: 10.1111/pbi.12154 (PMC4238783; doi:10.1111/pbi.12154)
Supplement: Figure S1 — Salix and Populus trichocarpa orthologous MAX protein alignments for MAX1-3. [file pbi0012-0480-SD1.pdf]

A)

```
                   1        10         20         30         40         50         60         70         80         90
PtrMAX1  MDLQVLFTDV PVVTAIICTV FAMLLGLLGY LYGPYWGVRK VPGPPVIPLL GHLPLMAKHG PDVFSVLAKR YGPIFRFHMG RQPLIIVADP
SxMAX1B  MDLQVLFTDV TMVTAIICTV SAMLLGLLGY LYGPYWGVRK VPGPPVFPLL GHLPLMAKHG PDVFSVLAKR YGPIFRFHMG RQPLIIVADP
SxMAX1C  MDLQVLFTDV TMVTAIICTV SAMLLGLLGY LYGPYWGVRK VPGPPVFPLL GHLPLMAKHG PDVFSVLAKR YGPIFRFHMG RQPLIIVADP
SxMAX1D  MDLQVLFTDV TMVMAIICTV SAMLLGLLGY LYGPYWGVRK VPGPPVFPLL GHLPLMAKHG PDVFSVLAKR YGPIFRFHMG RQPLIIVADP
SxMAX1E  MDLQVLFTDV TMVTAILCTV SAMLLGLLGY LYGPYWGVRK VPGPPVFPLL GHLPLMAKHG PDVFSVLAKR YGPIFRFHMG RQPLIIVADP
SxMAX1F  MDLQVLFTDV TMVTAIICTV SAVLLGLLGY LYGPYWGVRK VPGPPVFPLL GHLPLMAKHG PDVFSVLAKR YGPIFRFHMG RQPLIIVADP
SxMAX1G  MDLQVLFTDV TMVTAIICTV SAVLLGLLGY LYGPYWGVRK VPGPPVFPLL GHLPLMAKHG PDVFSVLAKR YGPIFRFHMG RQPLIIVADP
SxMAX1H  MDLQVLFTDV TMVTAIICTV SAMLLGLLGY LYGPYWGVRK VPGPPVFPLL GHLPLMAKHG PDVFSVLAKR YGPIFRFHMG RQPLIIVADP

                           100        110        120        130        140        150        160        170        180
PtrMAX1  ELCKEVAIKK FKDIPNRSVP SPISASPLHQ KGLFFTRDAR WSTMRNTILS VYQPSHLASL VPTMQSFIES ATDNFQSSNE EITFSNFSIK
SxMAX1B  ELCREVAIKK FKDIPNRSVP SPISASPLHQ KGLFFTRDAR WSTMRNTILS VYQPSHLASL VPTMQSYIES ATENFQSSNE EISFSNFSIK
SxMAX1C  ELCREVAIKK FKDIPNRSVP SPISASPLHQ KGLFFTRDAR WSTMRNTILS VYQPSHLASL VPTMQSYIES ATENFQSSNE EISFSNFSIK
SxMAX1D  ELCREVAIKK FKDIPNRSVP SPISASPLHQ KGLFFTRDAR WSTMRNTILS VYQPSHLASL VPTMQSYIES ATENFQSSNE EISFSNFSIK
SxMAX1E  ELCREVAIKK FKDIPNRSVP SPISASPLHQ KGLFFTRDAR WSTMRNTILS VYQPSHLASL VPTMQSYIES ATENFQSSNE EISFSNFSIK
SxMAX1F  ELCKEVAIKK FKDIPNRSVP SPISASPLHQ KGLFFTRDAR WSTMRNTILS VYQPSHLASL VPTMQSYIES ATENFQSSDE EISFSNFSIK
SxMAX1G  ELCKEVAIKK FKDIPNRSVP SPISASPLHQ KGLFFTRDAR WSTMRNTILS VYQPSHLASL VPTMQSYIES ATENFQSSDE EISFSNFSIK
SxMAX1H  ELCREVAIKK FKDIPNRSVP SPISASPLHQ KGLFFTRDAR WSTMRNTILS VYQPSHLASL VPTMQSYIES ATENFQSSNE EISFSNFSIK

                           190        200        210        220        230        240        250        260        270
PtrMAX1  LATDVIGQAA FGVDFGLSKP QSASDSINSF HNQGK-DNCD VSEFINQHIY STTQIKMDLS GSFSIITGLL VPILQEPFRQ IIKRIPGTMD
SxMAX1B  LATDVIGQAA FGVDFGLSKP QSASDSIKSF HNQGKEDNCD VSEFINQHIY STTQIKMDLS GSFSIILGLL VPILQEPFRQ IIKRIPGTMD
SxMAX1C  LATDVIGQAA FGVDFGLSKP QSASDSIKSF HNQGKEDNYD VSEFINQHIY STTQIKMDLS GSFSIILGLL VPILQEPFRQ IIKRIPGTMD
SxMAX1D  LATDVIGQAA FGVDFGLSKP QSASDSIKSF HNQGKEDNCD VSEFINQHIY STTQIKMDLS GSFSIILGLL VPILQEPFRQ IIKRIPGTMD
SxMAX1E  LATDVIGQAA FGVDFGLSKP QSASDSIKSF HNQDKEDNCD VSEFINQHIY STTQIKMDLS GSFSIILGLL VPILQEPFRQ IIKRIPGTMD
SxMAX1F  LATDVIGQAA FGVDFGLSKP QSASDTIKSF HNQGKEDNCD VSEFINQHIY STTQIKMDLS GSFSIILGLL VPILQEPFRQ IIKRIPGTMD
SxMAX1G  LATDVIGQAA FGVDFGLSKP QSASDTIKSF HNQGKEDNCD VSEFINQHIY STTQIKMDLS GSFSIILGLL VPILQEPFRQ IIKRIPGTMD
SxMAX1H  LATDVIGQAA FGVDFGLSKP QSASDSIKSF HNQDKEDNCD VSEFINQHIY STTQIKMDLS GSFSIILGLL VPILQEPFRQ IIKRIPGTMD

                           280        290        300        310        320        330        340        350        360
PtrMAX1  WKVDRTNRNI SGRLDEIVRK KMEEKNRGSK DFLSLIIRAR ESETLSKKVF TPDYISAVTY EHLLAGSITT SFTLSSVVYL VAQHPETEKK
SxMAX1B  WKVDRTNRDI SSRLDEIVRK KMEEKNRGSK DFLSLILHAR ESETLSKKVF SPDYISAVTY EHLLAGSVTT SFTLSSVVYL VAQHPETEKK
SxMAX1C  WKVDRTNRDI SSRLDEIVRK KMEEKNRGSK DFLSLILHAR ESESLSKKVF SPDYISAVTY EHLLAGSVTT SFTLSSVVYL VAQHPETEKK
SxMAX1D  WKVDRTNRDI SSRLDEIVRK KMEEKNRGSK DFLSLILHAR ESETLSKKVF SPDYISAVTY EHLLAGSVTT SFTLSSVVYL VAQHPETEKK
SxMAX1E  WKVDRTNRDI SSRLDEIVRK KMEEKNRGSK DFLSLILHAR ESETMSKKVF SPDYISAVTY EHLLAGSVTT SFTLSSVVYL VAQHPETEKK
SxMAX1F  WKVDRTNRDI SSRLDEIVRK KMEEKNRGSK DFLSLILHAR ESETLSKKVF SPDYISAVTY EHLLAGSVTT SFTLSSVVYL VAQHPETEKK
SxMAX1G  WKVDRTNRDI SSRLDEIVRK KMEEKNRGSK DFLSLILHAR ESETLSKKVF SPDYISAVTY EHLLAGSVTT SFTLSSVVYL VAQHPETEKK
SxMAX1H  WKVDRTNRDI SSRLDEIVRK KMEEKNRGSK DFLSLILHAR ESETMSKKVF SPDYISAVTY EHLLAGSVTT SFTLSSVVYL VAQHPETEKK

                           370        380        390        400        410        420        430        440        450
PtrMAX1  LLAEIDGFGP HEQIPTALDL QNKFPYLDQV VKEAMRFYVV SPLVARETSK EVEIGGYVLP KGTWIWLAPG VLAKDPKNFP EPDRFKPERF
SxMAX1B  LLAEIDGFGP HEQIPTAHDL QNKFPYLDQV VKEAMRFYVV SPLVARETSK EVEIGGYVLP KGTWIWLAPG VLAKDPKNFP EPDKFKPERF
SxMAX1C  LLAEIDGFGP HEQIPTAHDL QNKFPYLDQV VKEAMRFYVV SPLVARETSK EVEIGGYVLP KGTWIWLAPG VLAKDPKNFP EPDKFKPERF
SxMAX1D  LLAEIDGFGP HEQIPTAHDL QNKFPYLDQV VKEAMRFYVV SPLVARETSK EVEIGGYVLP KGTWIWLAPG VLAKDPKNFP EPDKFKPERF
SxMAX1E  LLAEIDGFGP HEQIPTAHDL QNKFPYLDQV VKEAMRFYVV SPLVARETSK EVEIGGYVLP KGTWIWLAPG VLAKDPKNFP EPDKFKPERF
SxMAX1F  LLAEIDAFGP PEQIPTAHDL QNKFPYLDQV VKEAMRFYVV SPLVARETSK KVEIGGYVLP KGTWIWLAPG VLAKDPKNFP EPEKFKPERF
SxMAX1G  LLAEIDAFGP PEQIPTAHDL QNKFPYLDQV VKEAMRFYVV SPLVARETSK KVEIGGYVLP KGTWIWLAPG VLAKDPKNFP EPDKFKPERF
SxMAX1H  LLAEIDGFGP HEQIPTAHDL QNKFPYLDQV VKEAMRFYVV SPLVARETSK EVEIGGYVLP KGTWIWLAPG VLAKDPKNFP EPDKFKPERF

                           460        470        480        490        500        510        520        531
PtrMAX1  DPNCEEEKRR HPCALIPFGI GPRACIGKKF SIQEIKLSLI HLYRKYLFRH SPTMEKPIEF EFGIVINFKR GVKLRIIKRT *
SxMAX1B  DPNCEEEKRR HPYALIPFGI GPRACIGKKF SIQEIKLSLI HLYRKYVFRH SPDMEKPVEF EFGIVINFKH GVKLRIIKRT *
SxMAX1C  DPNCEEEKRR HPYALIPFGI GPRACIGKKF SIQEIKLSLI HLYRKYVFRH SPDMEKPVEF EFGIVINFKH GVKLRIIKRT *
SxMAX1D  DPNCEEEKRR HPYALIPFGI GPRACIGKKF SIQEIKLSLI HLYRKYVFRH SPDMEKPVEF EFGIVINFKH GVKLRIIKRT *
SxMAX1E  DPNCEEEKRR HPYALIPFGI GPRACIGKKF SIQEIKLSLI HLYRKYVFRH SPDMEKPVEF DFGIVINFKH GVKLRIIKRT *
SxMAX1F  DPNCEEEKRR HPYALIPFGI GPRACIGKKF SIQEIKLSLI HLYRKYVFRH SPDMEKPVEF EFGIVINFKH GVKLRIVKRT *
SxMAX1G  DPNCEEEKRR HPYALIPFGI GPRACIGKKF SIQEIKLSLI HLYRKYVFRH SPDMEKPVEF EFGIVINFKH GVKLRIVKRT *
SxMAX1H  DPNCEEEKRR HPYALIPFGI GPRACIGKKF SIQEIKLSLI HLYRKYVFRH SPDMEKPVEF DFGIVINFKH GVKLRIIKRT *
```

B)

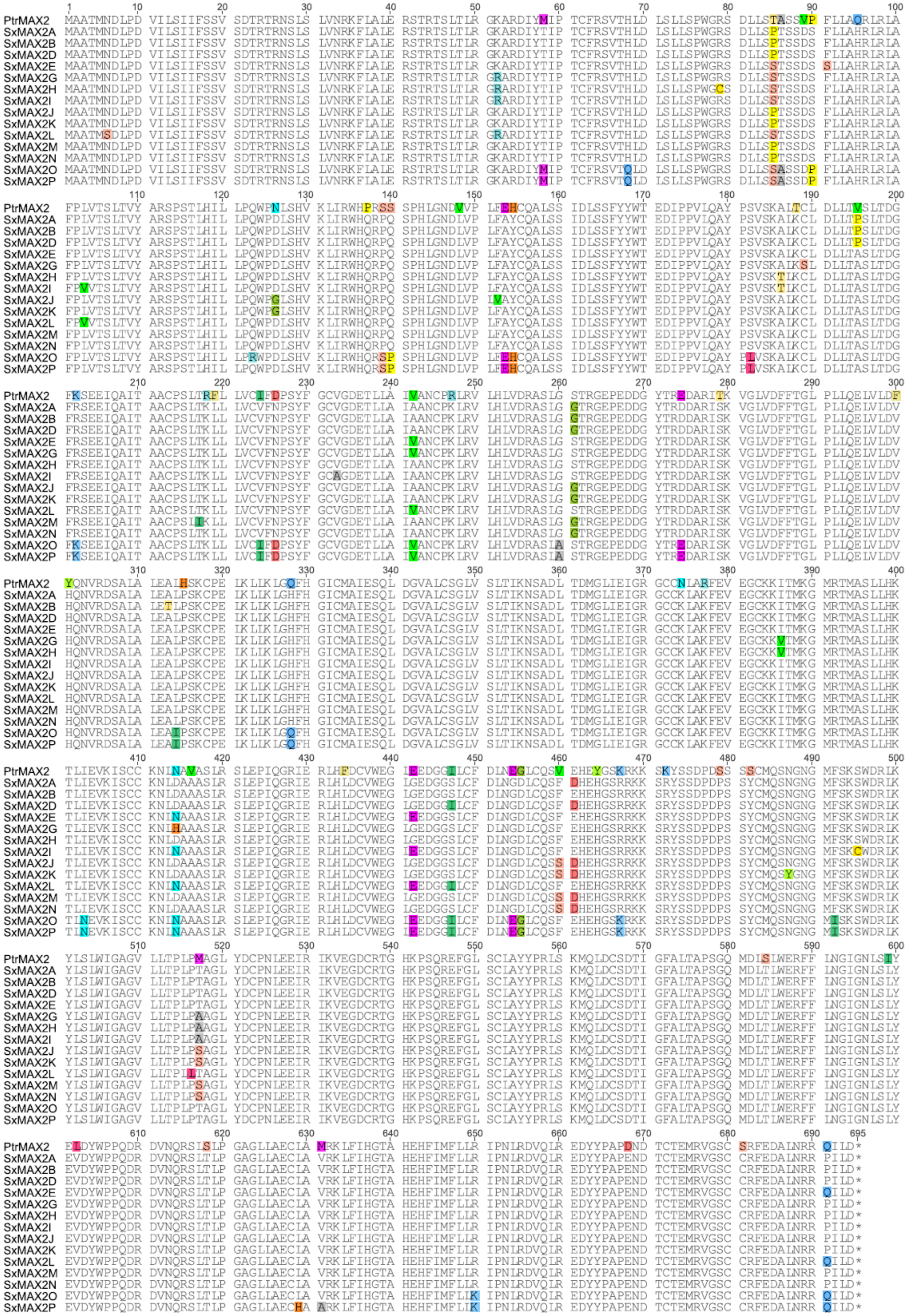

C)

```
          1        10         20         30         40         50         60         70         80         90
PtrMAX3*  MQARPYHTA- PTSFPSPSKL PRFHRTPSPA KIKIPRVISI STPDTADHAS S-RTSLDTID DHVAAFWDYQ FLFVSQRSET TEAVTLRVVD
SxMAX3A   MQAKPCHILI PTDFPSPIKL PRFHRIPSPA KIKIPRVAPI STPDSTSHAP GRRTALETVD DSVAAFWDYQ FLFVSQRSET AEAETLRVID
SxMAX3D   MQAKPCHILI PTDFPSPIKL PRFHRIPSPA KIKIPRVASI STPDSTSHAP GRRTALETVD DSVAAFWDYQ FLFVSQRSET AEAETLRVID
SxMAX3E   MQAKPCHVL- PTDFPSPIKL PRFHRIPSPA KIKIPRVASI STPDSTSHGP G-RTALETVD DSVAAFWDYQ FIFVSQRSET AEAETLRVID
SxMAX3F   MQAKPCHVLI PTDFPSPIKL PRFHRIPSPA KIKIPRVASI STPDSTSHAP GRRTALETVD DSVAAFWDYQ FLFVSQRSET AEAETLRVID
SxMAX3G   MQANPCHILI PTDFPSPIKL PRFHRIPSPA KIKIPRVAPI STPDSTSHAP GRRTALETVD DSVAAFWDYQ FLFVSQRSET AEAETLRVID
SxMAX3H   MQAKPCHLLI PTDFPSPIKL PRFHRIPSPA KIKIPRVASI STPDSTSHAP GRRTALETVD DSVAAFWDYQ FLFVSQRSET AEAETLRVID
SxMAX3I   MQAKPCHVLI PTDFPSPIKL PRFHRIPSPA KIKIPSVASI STPDSTSHAP GRRTALETVD DSVAAFWDYQ FLFVSQRSET AEAETLRVID
SxMAX3J   MQAKPCHVL- PTDFPSPIKL PRFHRIPSPA KIKIPRVASI STPDSTSHGP G-RTALETVD DSVAAFWDYQ FLFVSQRSET AEAETLRVID
SxMAX3K   MQAKPCHILI PTDFPSPIKL PRFHRIPSPA KIKIPRVASI STPDSTSHAP GRRTALETVD DSVAAFWDYQ FLFVSQRSET AEAETLRVID

                 100        110        120        130        140        150        160        170        180
PtrMAX3*  GAIPSNFPSG TYYLTGPGLF TDDHGSTVHP LDGHGYLRAF NIDGVSREVK FMARYVKTEA QVEEHDPETN TWRFTYRGPF SVIKGGKRLG
SxMAX3A   GAIPSDFPSG TYYLTGPGLF ADDHGSTVHP LDGHGYLRAF RIDGVSREVR FMARYVKTEA QVEEHDPETN SWRFTHRGPF SVIKGGKRLG
SxMAX3D   GAIPSDFPSG TYYLTGPGLF ADDHGSTVHP LDGHGYLRAF RIDAVSREVR FMARYVKTEA QVEEHDPETN SWRFTHRGPF SVIKGGKRLG
SxMAX3E   GAIPSDFPSG TYYLTGPGLF ADDHGSTVHP LDGHGYLRAF RIDGGSREVK FMARYVRTEA QAEEHDPETN SWRFTHRGPF SVIKGGKRLG
SxMAX3F   GAIPSDFPSG TYYLTGPGLF ADDHGSTVHP LDGHGYLRAF RIDAVSREVR FMARYVKTEA QVEEHDPETN SWRFTHRGPF SVIKGGKRLG
SxMAX3G   GAIPSDFPSG TYYLTGPGLF ADDHGSTVHP LDGHGYLRAF RIDAVSREVR FMARYVKTEA QVEEHDPETN SWRFTHRGPF SVIKGGKRLG
SxMAX3H   GAIPSDFPSG TYYLTGPGLF ADDHGSTVHP LDGHGYLRAF RIDAVSREVR FMARYVKTEA QVEEHDPETN SWRFTHRGPF SVIKGGKRLG
SxMAX3I   GAIPSDFPSG TYYLTGPGLF ADDHGSTVHP LDGHGYLRAF RIDAVSREVK FMARYVKTEA QVEEHDPETN SWRFTHRGPF SVIKGGKRLG
SxMAX3J   GAIPSDFPSG TYYLTGPGLF ADDHGSTVHP LDGHGYLRAF RIDGVSREVK FMARYVKTEA QVEEHDPETN SWRFTHRGPF SVIKGGKRLG
SxMAX3K   GAIPSDFPSG TYYLTGPGLF ADDHGSTVHP LDGHGYLRAF RIDGVSREVR FMARYVKTEA QVEEHDPETN SWRFTHRGPF SIIKGGKRLG

                 190        200        210        220        230        240        250        260        270
PtrMAX3*  NTKVMKNVAN TSVLRWGGKL LCLWEGGDPY EIEIGTLDTT GRFD-MINGR DSSMDNRSNG GDTWDIAAGL IKPILHGVFK MPPKRLLSHY
SxMAX3A   NVKVMKNVAN TSVLRWGGKL LCLWEGGDPY EIEPGTLDTT GRFD-MIDGC DSLMDNRSSC GDPWDIAAGL IKPILHGVFK MPPKRLLSHY
SxMAX3D   NVKVMKNVAN TSVLRWGGKL LCLWEGGDPY EIEPGTLDTT GRFDHMIDGC DSLMDNRSSC GDPWDIAAGL IKPILHGVFK MPPKRLLSHY
SxMAX3E   NVKVMKNVAN TSVLRWGGKL LCLWEGGDPY EIEPGTLDTT GRFD-MIDGC DSLMDNRSNC GDPWDIAAGL IKPILHGVFK MPPKRLLSHY
SxMAX3F   NVKVMKNVAN TSVLRWGGKL LCLWEGGDPY EIEPGTLDTT GRFDHMIDGC DSLMDNRSNC GDPWDIAAGL IKPILHGVFK MPPKRLLSHY
SxMAX3G   NVKVMKNVAN TSVLRWGGKL LCLWEGGDPY EIEPGTLDTT GRFDHMIDGC DSLMDNRSNC GDPWDIAAGL IKPILHGVFK MPPKRLLSHY
SxMAX3H   NVKVMKNVAN TSVLRWGGKL LCLWEGGDPY EIEPGTLDTT GRFDHMIDGC DSLMDNRSNC GDPWDIAAGL IKPILHGVFK MPPKRLLSHY
SxMAX3I   NVKVMKNVAN TSVLRWGGKL LCLWEGGDPY EIEPGTLDTT GRFDHMIDGC DSLMDNRSNC GDPWDIAAGL IKPILHGVFK MPPKRLLSHY
SxMAX3J   NVKVMKNVAN TSVLRWGGKL LCLWEGGDPY EIEPGTLDTT GRFDHMIDGC DSLMDNRSSC GDPWDIAAGL IKPILHGVFK MPPKRLLSHY
SxMAX3K   NVKVMKNVAN TSVLRWGGKL LCLWEGGDPY EIEPGTLDTT GRFD-MIDGC DSLMDNRSSC GDPWDIAAGL IKPILHGVFK MPPKRLLSHY

                 280        290        300        310        320        330        340        350        360
PtrMAX3*  KIDAKRNRLI TASCNAEDML LPRSNFTFYE YDENFKLLQR QEFNIPDHIM IHDWAFTDTH YILFANRIKL DVIGSMTAIC GLSPMISALS
SxMAX3A   KIDVKSHRLI TTSCNAEDML LPRSNFTFYE YDENFKLLQR QEFNIPDHIM IHDWAFTDTH YILFANRIKL DVIGSMAAVC GLSPMISALS
SxMAX3D   KIDVKSHRLI TTSCNAEDML LPRSNFTFYE YDENFKLLQR QEFNIPDHIM IHDWAFTDSH YILFANRIKL DVIGSMAAVC GLSPMISALS
SxMAX3E   KIDAKSHRLI TTSCNAEDML LPRSNFTFYE YDENFKLLQR QEFNIPDHIM IHDWAFTDTH YILFANRIKL DVIGSMAAVC GLSPMISALS
SxMAX3F   KIDAKRHRLI TTSCNAEDML LPRSNFTFYE YDENFKLLQR QEFNIPDHIM IHDWAFTDTH YILFANRIKL DVIGSMAAVC GLSPMISALS
SxMAX3G   KIDVKSHRLI TTSCNAEDML LPRSNFTFYE YDENFKLLQR QEFNIPDHIM IHDWAFTDTH YILFANRIKL DVIGSMAAVC GLSPMISALS
SxMAX3H   KIDAKRHRLI TTSCNAEDML LPRSNFTFYE YDENFKLLQR QEFNIPDHIM IHDWAFTDTH YILFANRIKL DVIGSMAAVC GLSPMISALS
SxMAX3I   KIDAKRHRLI TTSCNAEDML LPRSNFTFYE YDENFKLLQR QEFNIPDHIM IHDWAFTDTH YILFANRIKL DVIGSMAAVC GLSPMISALS
SxMAX3J   KIDAKRHRLI TTSCNAEDML LPRSNFTFYE YDENFKLLQR QEFNIPDHIM IHDWAFTDTH YILFANRIKL DVIGSMAAVC GLSPMISALS
SxMAX3K   KIDVKSHRLI TTSCNAEDML LPRSNFTFYE YDENFKLLQR QEFNIPDHIM IHDWAFTDTH YILFANRIKL DVIGSMAAVC GLSPMISALS

                 370        380        390        400        410        420        430        440        450
PtrMAX3*  VNPSKSTSPI YLLPRFPEKS PSNRNWRVPV EAPSTMWLLH VGNAYEAKDV HGNLQIQIHA SICSYQWFNF QKLFGYNWKN AKLDPSVMNV
SxMAX3A   VNPSKSTSPI YLLPRFPGKS SSNRNWRVPV EAPSTMWLLH VGNAYEAKDV NGNLQIQIHA SICSYQWFNF KKLFGYDWQN NKLDPSIMNV
SxMAX3D   VNPSKSTSPI YLLPRFPGKS SSNRNWRVPV EAPSTMWLLH VGNAYEAKDV NGNLQIQIHA SICSYQWFNF KKLFGYDWQN NKLDPSIMNV
SxMAX3E   VNPSKSTSPI YLLPRFPGKS SSNRNWRVPV EAPSTMWLLH VGNAYEAKDV NGNLQIQIHA SICSYQWFNF KKLFGYDWQN NKLDPSIMNV
SxMAX3F   VNPSKSTSPI YLLPRFPGKS SSNRNWRVPV EAPSTMWLLH VGNAYEAKDV NGNLQIQIHA SICSYQWFNF KKLFGYDWQN NKLDPSIMNV
SxMAX3G   VNPSKSTSPI YLLPRFPGKS SSNRNWRVPV EAPSTMWLLH VGNAYEAKDV NGNLQIQIHA SICSYQWFNF KKLFGYDWQN NKLDPSIMNV
SxMAX3H   VNPSKSTSPI YLLPRFPGKS SSNRNWRVPV EAPSTMWLLH VGNAYEAKDV NGNLQIQIHA SICSYQWFNF KKLFGYDWQN NKLDPSIMNV
SxMAX3I   VNPSKSTSPI YLLPRFPGKS SSNRNWRVPV EAPSTMWPLH VGNAYEAKDV NGNLQIQIHA SICSYQWFNF KKLFGYDWQN NKLDPSIMNV
SxMAX3J   VNPSKSTSPI YLLPRFPGKS SSNRNWRVPV EAPSTMWLLH VGNAYEAKDV NGNIHIQIHA SICSYQWFNF KKLFGYDWQN NKLDPSIMNV
SxMAX3K   VNPSKSTSPI YLLPRFPGKS SSNRNWRVPV EAPSTMWLLH VGNAYEAKDV NGNLQIQIHA SICSYQWFNF KKLFGYDWQN NKLDPSIMNV

                 460        470        480        490        500        510        520        530        540
PtrMAX3*  KEGGDELLPH LVQVSINLDA DGTCQESSVE PLNQWSKPAD FPVINPDFSG NKNKYVYAAS SSGSRQTLPH FPFDMVVKIN ILDKSIHTWT
SxMAX3A   KEGGDELLPR LVQVYINLDA DGTCQKSSVE PLNQWSKTTD FPVINPDFSG NKNKYVYAAS SSGSRQTLPH FPFDTVVKLD LSDKSIHTWT
SxMAX3D   KEGGDELLPR LVQVYINLDA DGTCQKSSVE PLNQWSKTTD FPVINPDFSG NKNKYVYAAS SSGSRQTLPH FPFDTVVKLD LSDKSIHTWT
SxMAX3E   KEGGDELLPR LVQVYINLDA DGTCQKSSVE PLNQWSKTTD FPVINPDFSG NKNKYVYAAS SSGSRQTLPH FPFDTVVKLD LSDKSIHTWT
SxMAX3F   KEGGDELLPR LVQVYINLDA DGTCQKSSVE PMNQWSKTTD FPVINPDFSG NKNKYVYAAS SSGSRQTLPH FPFDTVVKLD LSDKSIHTWT
SxMAX3G   KEGGDELLPR LVQVYINLDA DGTCQKSSVE PLNQWSKTTD FPVINPDFSG NKNKYVYAAS SSGSRQTLPH FPFDTVVKLD LSDKSIHTWT
SxMAX3H   KEGGDELLPR LVQVYINLDA DGTCQKSSVE PMNQWSKTTD FPVINPDFSG NKNKYVYAAS SSGSRQTLPH FPFDTVVKLD LSDKSIHTWT
SxMAX3I   KEGGDELLPR LVQVYINLDA DGTCQKSSVE PLNQWSKTTD FPVINPDFSG NKNKYVYAAS SSGSRQTLPH FPFDTVVKLD LSDKSIHTWT
SxMAX3J   KEGGDELLPR LVQVYINLDA DGTCQKSSVE PLNQWSKTTD FPVINPDFSG NKNKYVYAAS SSGSRQTLPH FPFDTVVKLD LSDKSIHTWT
SxMAX3K   KEGGDELLPR LVQVYINLDA DGTCQKSSVE PLNQWSKTTD FPVINPDFSG NKNKYVYAAS SSGSRQTLPH FPFDTVVKLD LSDKSIHTWT

                 550        560        570        580        590        600        610      617
PtrMAX3*  VGARRFIGEP IFVPKGREED DGYLLVVEYA VAIQRCYLVI LNPKRIGKAD ALVARLEVPR HINFPLGFHG FWANGS*
SxMAX3A   VGARRFIGEP IFVSNGREED DGYLLVVEYA VAIQRCYLVI LNPKRIGKAD ALVARLEVPK HINFPLGFHG FWAKDI*
SxMAX3D   VGARRFIGEP IFVSNGREED DGYLLVVEYA VAIQRCYLVI LNPKRIGKAD ALVARLEVPK HINFPLGFHG FWAKDI*
SxMAX3E   VGARRFIGEP IFVSNGREED DGYLLVVEYA VAIQRCYLVI LNPKRIGKAD ALVARLEVPK HINFPLGFHG FWAKDI*
SxMAX3F   VGARRFIGEP VFVSNGREED DGYLLVVEYA VAIQRCYLVI LNPKRIGKAD ALVARLEVPK HINFPLGFHG FWAKDI*
SxMAX3G   VGARRFIGEP IFVSNGREED DGYLLVVEYA VAIQRCYLVI LNPKRIGKAD ALVARLEVPK HINFPLGFHG FWAKDI*
SxMAX3H   VGARRFIGEP IFVSNGREED DGYLLVVEYA VAIQRCYLVI LNPKRIGKAD ALVARLEVPK HINFPLGFHG FWAKDI*
SxMAX3I   VGARRFIGEP IFVSNGREED DGYLLVVEYA VAIQRCYLVI LNPKRIGKAD ALVARLEVPK HINFPLGFHG FWAKDI*
SxMAX3J   VGARRFIGEP IFVSNGREED DGYLLVVEYA VAIQRCYLVI LNPKRIGKAD ALVARLEVPK HINFPLGFHG FWAKDI*
SxMAX3K   VGARRFIGEP IFVSNGREED DGYLLVVEYA VAIQRCYLVI LNPKRIGKAD ALVARLEVPK HINFPLGFHG FWAKDI*
```
