# Supplementary material for: Functional screening of willow alleles in Arabidopsis combined with QTL mapping in willow (Salix) identifies SxMAX4 as a coppicing response gene
Source: Plant Biotechnol J. 2014 Jan 7;12(4):480–91. doi: 10.1111/pbi.12154 (PMC4238783; doi:10.1111/pbi.12154)
Supplement: Table S1 — Salix MAX cloning information. [file pbi0012-0480-SD2.pdf]

**Supplementary Table S1.** *Salix MAX* cloning information.

| Allele | F-Primer† | R-Primer | DNA Source |
|---|---|---|---|
| ***SxMAX1*** | | | |
| S3 (B) | ATGGATTTACAGGTTTTGTTTACAG | TCAAGTTCGTTTTATGATTCTAAGC | S3 |
| R13.2 (C) | ATGGATTTACAGGTTTTGTTTACAG | TCAAGTTCGTTTTATGATTCTAAGC | R13 |
| RES0453 (D) | CGAGCATCCATCTACTTCGAG | CACGATAGGGCTAGATACGTG | RES0453 |
| RES0789.2 (E) | CGAGCATCCATCTACTTCGAG | CACGATAGGGCTAGATACGTG | RES0789 |
| RES0099.1 (F) | CGAGCATCCATCTACTTCGAG | CACGATAGGGCTAGATACGTG | RES0099 |
| RES0099.2 (G) | CGAGCATCCATCTACTTCGAG | CACGATAGGGCTAGATACGTG | RES0099 |
| RES0901.1 (H) | CGAGCATCCATCTACTTCGAG | CACGATAGGGCTAGATACGTG | RES0901 |
| ***SxMAX2*** | | | |
| S3 (A) | ATGGCTGCTACCATGAACGATC | TCAGTCGAGGATCGGACGCC | S3 |
| R13.2 (B) | ATGGCTGCTACCATGAACGATC | TCAGTCGAGGATCGGACGCC | R13 |
| RES0674.2 (D) | ATGGCTGCTACCATGAACGATC | TCAGTCGAGGATCGGACGCC | RES0674 |
| RES0789.1 (E) | GGTTGAATAGCTAGGATGGC | TGCTCTATGAGGAACAGGCTAG | RES0789 |
| RES0432.1 (G) | GGTTGAATAGCTAGGATGGC | TGCTCTATGAGGAACAGGCTAG | RES0432 |
| RES0432.2 (H) | GGTTGAATAGCTAGGATGGC | TGCTCTATGAGGAACAGGCTAG | RES0432 |
| RES0453 (I) | GGTTGAATAGCTAGGATGGC | TGCTCTATGAGGAACAGGCTAG | RES0453 |
| RES0615.1 (J) | GGTTGAATAGCTAGGATGGC | TGCTCTATGAGGAACAGGCTAG | RES0615 |
| RES0615.2 (K) | GGTTGAATAGCTAGGATGGC | TGCTCTATGAGGAACAGGCTAG | RES0615 |
| RES1059.2 (L) | GGTTGAATAGCTAGGATGGC | TGCTCTATGAGGAACAGGCTAG | RES1059 |
| RES0627.2 (M) | GGTTGAATAGCTAGGATGGC | TGCTCTATGAGGAACAGGCTAG | RES0627 |
| RES0628.2 (N) | GGTTGAATAGCTAGGATGGC | TGCTCTATGAGGAACAGGCTAG | RES0628 |
| RES0099.1 (O) | GGTTGAATAGCTAGGATGGC | TGCTCTATGAGGAACAGGCTAG | RES0099 |
| RES0099.2 (P) | GGTTGAATAGCTAGGATGGC | TGCTCTATGAGGAACAGGCTAG | RES0099 |
| ***SxMAX3*** | | | |
| R13.2 (A) | ATGCAGGCAAAACCATGCC | TTAAATATCTTTGGCCCAAAAACC | R13 |
| RES0655 (D) | ATGCAGGCAAAACCATGCC | TTAAATATCTTTGGCCCAAAAACC | RES0655 |
| RES0789.1 (E) | ATGCAGGCAAAMCCATGCC | TTAAATATCTTTGGCCCAAAAACC | RES0789 |
| RES0628.2 (F) | ATGCAGGCAAAACCATGCC | TTAAATATCTTTGGCCCAAAAACC | RES0628 |
| RES0789.2 (G) | ATGCAGGCAAAMCCATGCC | TTAAATATCTTTGGCCCAAAAACC | RES0789 |
| RES0627.2 (H) | CTCGTGACAACTTACCACACAC | TTAAATATCTTTGGCCCAAAAACC | RES0627 |
| RES0615.1 (I) | ATGCAGGCAAAACCATGCC | TTAAATATCTTTGGCCCAAAAACC | RES0615 |
| RES0506.1 (J) | CTCGTGACAACTTACCACACAC | TTAAATATCTTTGGCCCAAAAACC | RES0506 |
| RES0901.1 (K) | CTCGTGACAACTTACCACACAC | TTAAATATCTTTGGCCCAAAAACC | RES0901 |
| ***SxMAX4*** | | | |
| S3 (B) | ATGGCTTCCTTGGCATTTTCC | TTATTTCTTTGGCACCCAGCATC | S3 |
| RES0674.2 (D) | CTCCAACTTGGTATGTCTCCC | GATAGCTAAATCACACAACCCC | RES0674 |
| RES0453.1 (E) | CTCCAACTTGGTATGTCTCCC | GATAGCTAAATCACACAACCCC | RES0453 |
| RES0453.2 (F) | CTCCAACTTGGTATGTCTCCC | GATAGCTAAATCACACAACCCC | RES0453 |
| RES0789.1 (G) | CTCCAACTTGGTATGTCTCCC | GATAGCTAAATCACACAACCCC | RES0789 |
| RES0789.2 (H) | CTCCAACTTGGTATGTCTCCC | GATAGCTAAATCACACAACCCC | RES0789 |
| RES0901.1 (J) | CTCCAACTTGGTATGTCTCCC | GATAGCTAAATCACACAACCCC | RES0901 |
| RES0901.2 (K) | CTCCAACTTGGTATGTCTCCC | GATAGCTAAATCACACAACCCC | RES0901 |
| RES1059.2 (L) | CTCCAACTTGGTATGTCTCCC | GATAGCTAAATCACACAACCCC | RES1059 |
| RES0506.1 (M) | CTCCAACTTGGTATGTCTCCC | GATAGCTAAATCACACAACCCC | RES0506 |
| RES0506.2 (N) | CTCCAACTTGGTATGTCTCCC | GATAGCTAAATCACACAACCCC | RES0506 |
| RES0099.1 (O) | CTCCAACTTGGTATGTCTCCC | GATAGCTAAATCACACAACCCC | RES0099 |
| RES0099.2(Q) | CTCCAACTTGGTATGTCTCCC | GATAGCTAAATCACACAACCCC | RES0099 |
| RES0615.1 (R) | CTCCAACTTGGTATGTCTCCC | GATAGCTAAATCACACAACCCC | RES0615 |
| RES0615.2 (S) | CTCCAACTTGGTATGTCTCCC | GATAGCTAAATCACACAACCCC | RES0615 |
| RES0628.2 (T) | CTCCAACTTGGTATGTCTCCC | GATAGCTAAATCACACAACCCC | RES0628 |
| RES0627.1 (U) | CTCCAACTTGGTATGTCTCCC | GATAGCTAAATCACACAACCCC | RES0627 |

**†***CACC* was added to the start of every forward primer for directional cloning purposes.
